# Improved black-blood imaging using DANTE-SPACE for combined carotid and intracranial vessel wall evaluation

**DOI:** 10.1186/1532-429X-17-S1-O17

**Published:** 2015-02-03

**Authors:** Yibin Xie, Qi Yang, Zhaoyang Fan, Debiao Li

**Affiliations:** 1 Department of Bioengineering, University of California, Los Angeles, Los Angeles, CA, USA; 2 Department of Biomedical Sciences, Cedars-Sinai Medical Center, Los Angeles, CA, USA; 3 Departments of Radiology, Xuanwu Hospital, Capital Medical University, Beijiang, China

## Background

Variable flip angle 3D TSE (SPACE) is a promising technique for vessel wall imaging that provides high resolution and the freedom of 3D retrospective formatting compared with 2D TSE. However it is difficult to completely suppress residual luminal blood which may mimic plaque leading to difficulties in diagnosis [Fan. JMRI. 2010:31], especially after contrast enhancement (CE). Additionally, a more efficient imaging protocol is needed that covers both carotid and intracranial arteries as atherosclerosis is a systematic disease that usually affects multiple vascular beds. The purpose of this work is to develop a 3D combined carotid and intracranial vessel wall imaging protocol with high resolution (0.7mm^3^ isotropic) and excellent blood suppression both pre- and post-CE.

## Methods

A DANTE module [Li. MRM. 2012:68] was added before each SPACE readout train in order to improve the suppression of slow flow. The DANTE pulse train parameters were optimized based on simulations and phantom study to minimize SNR loss and banding artifacts while maintaining sufficient flow dephasing effects. A 16-channel head/neck coil was used together with a 4-channel special purpose surface coil on a 3T scanner (Siemens Verio). A coronal imaging slab slightly rotated transversely was oriented to cover both carotid and vertibrobasilar arterial systems with one scan. Healthy subjects (n=14) and symptomatic patients (n=6) were scanned with DANTE-SPACE, SPACE-only and T1w 2D TSE in a randomized order pre- and post-CE (0.1mmol/kg). DANTE-SPACE parameters include: FA = 10; train length = 100; G_xyz_ = 25 mT/m; TR = 780ms; spatial resolution = 0.7mm^3^ isotropic; 80 slices; scan time = 5'57".

## Results

**Figure 1:**
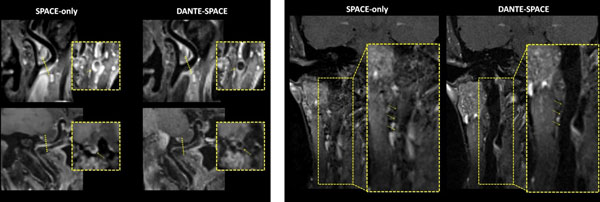
**(A)** Two representative examples of improved arterial blood suppression by DANTE-SPACE post-CE. On the SPACE-only images significant residual blood was observed at internal carotid artery, which obscured the boundary between lumen and vessel wall and may be misidentified as a plaque (yellow arrow). On the DANTE-SPACE images lumen was clean and vessel wall appeared thin. **(B)** A representative example of improved venous blood suppression by DANTE-SPACE. On the post-CE DANTE-SPACE images the venous blood was well suppressed and the venous lumen appeared dark (arrows). Also note the reduced level of flow artifacts compared to SPACE only images.

**Figure 2:**
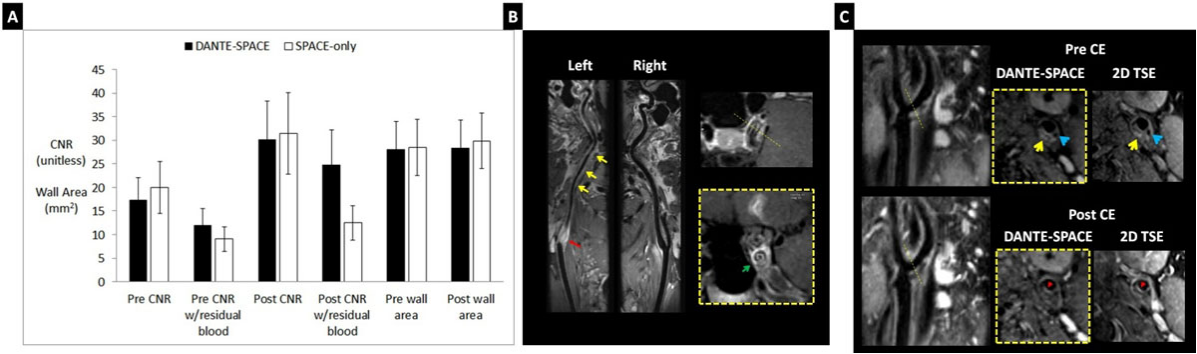
**(A)** Vessel wall CNR and wall area measurements comparison between DANTE-SPACE and SPACE-only. DANTE-SPACE had significantly higher CNR than SPACE-only when residual blood was present. **(B)** Post-CE DANTE-SPACE images of a patient suspected of ICA dissection. Note the thrombus near the bifurcation of the left internal carotid artery (**red arrow**) and thickened arterial wall (**yellow arrows**). In-plane and cross-sectional images showed double lumen sign (**green arrow**) which confirmed the diagnosis of dissection. **(C)** Pre- and post-CE DANTE-SPACE images from a symptomatic patient with slice-matched T1w 2D TSE images as reference. Pre-CE images at the bifurcation showed hyperintensive plaque component suggesting intraplaque hemorrhage (**yellow arrows**). Also note the hypointense plaque component suggesting calcification (**blue arrows**). Post-CE images showed enhanced plaque component next to the lumen suggesting thin fibrous cap (**red arrows**).

DANTE-SPACE improved both arterial and venous blood suppression compared with SPACE-only (Figure 1). Five (pre-CE) and 9 (post-CE) out of 14 healthy subjects had apparent residual blood with SPACE-only whereas 2 (post-CE) subject had apparent residual blood with DANTE-SPACE. Blinded region-of-interest analysis showed 33% (pre-CE) and 98% (post-CE) improvement in wall CNR with DANTE-SPACE over SPACE-only when residual blood was present (both p<0.001) (Figure 2A). Also vessel wall area measured from SPACE-only images was significantly larger (5.2%, p<0.001) than that from DANTE-SPACE images, possibly caused by incomplete blood suppression. Example images from the patient study were shown in Figure 2B where DANTE-SPACE helped diagnose a patient with internal carotid artery dissection and in Figure 2C where DANTE-SPACE helped identify a vulnerable plaque with calcification, intra-plaque haemorrhage and thin fibrous cap.

## Conclusions

DANTE-SPACE significantly improved blood suppression and vessel wall CNR both pre- and post-CE when SPACE-only resulted in residual blood. Combined high-resolution carotid and intracranial imaging using DANTE-SPACE was feasible and time-efficient (<6 min), with the potential ability to identify different plaque components including calcification, intra-plaque haemorrhage and fibrous cap.

## Funding

NIH/NHLBI: R01HL096119.

